# The Interplay of LncRNA-H19 and Its Binding Partners in Physiological Process and Gastric Carcinogenesis

**DOI:** 10.3390/ijms18020450

**Published:** 2017-02-20

**Authors:** Li Zhang, Yuhang Zhou, Tingting Huang, Alfred S. L. Cheng, Jun Yu, Wei Kang, Ka Fai To

**Affiliations:** 1 Department of Anatomical and Cellular Pathology, State Key Laboratory of Oncology in South China, Prince of Wales Hospital, The Chinese University of Hong Kong, Hong Kong, China; li.3.zhang@uconn.edu (L.Z.); zyhjoe@gmail.com (Y.Z.); huangtingting0531@gmail.com (T.H.); 2 Institute of Digestive Disease, Partner State Key Laboratory of Digestive Disease, The Chinese University of Hong Kong, Hong Kong, China; alfredcheng@cuhk.edu.hk (A.S.L.C.); junyu@cuhk.edu.hk (J.Y.); 3 Li Ka Shing Institute of Health Science, Sir Y.K. Pao Cancer Center, The Chinese University of Hong Kong, Hong Kong, China; 4 Shenzhen Research Institute, The Chinese University of Hong Kong, Hong Kong, China; 5 School of Biomedical Sciences, The Chinese University of Hong Kong, Hong Kong, China; 6 Department of Medicine and Therapeutics, The Chinese University of Hong Kong, Hong Kong, China

**Keywords:** lncRNA, H19, gastric cancer

## Abstract

Long non-coding RNA (lncRNA), a novel and effective modulator in carcinogenesis, has become a study hotspot in recent years. The imprinted oncofetal lncRNA H19 is one of the first identified imprinted lncRNAs with a high expression level in embryogenesis but is barely detectable in most tissues after birth. Aberrant alterations of H19 expression have been demonstrated in various tumors, including gastric cancer (GC), implicating a crucial role of H19 in cancer progression. As one of the top malignancies in the world, GC has already become a serious concern to public health with poor prognosis. The regulatory roles of H19 in gastric carcinogenesis have been explored by various research groups, which leads to the development of GC therapy. This review comprehensively summarizes the current knowledge of H19 in tumorigenesis, especially in GC pathogenesis, with emphasis on the underneath molecular mechanisms depicted from its functional partners. Furthermore, the accumulated knowledge of H19 will provide better understanding on targeted therapy of GC.

## 1. Introduction

Long non-coding RNAs (lncRNAs) are a group of regulatory RNAs longer than 200 nucleotides with no protein-coding potential. A large number of lncRNAs have been identified from mammalian genome within recent years. According to the genomic localization, lncRNAs can be catalogued roughly into intergenic, intronic, bidirectional, enhancer, sense and antisense lncRNAs [1]. Up to now, most of the studies about lncRNAs focus on their regulatory role on gene expression at both transcriptional and post-transcriptional levels [2]. Despite limited conservation in terms of primary sequence, lncRNAs function similarly in different species. For instance, lncRNAs usually exert their functions through interacting with distinct partners including proteins and miRNAs as decoyer, guider or scaffold [3].

Four common molecular behaviors of lncRNAs have been summarized [3]. Firstly, they convey signals. LncRNAs are sometimes considered as signal markers of some biological events, because they show special expression patterns under certain physiological or pathological conditions [4]. Secondly, some lncRNAs are also known as decoys, which function as “molecular sink” to titrate away RNA or protein targets [5,6]. The third point is that there are studies suggesting lncRNAs to be guiders [3,7,8]. This subtype of lncRNAs binds to proteins and leads the ribonucleoproteins (RNPs) to target sites to convey regulation information. Moreover, previous research also demonstrated the role of some lncRNAs as scaffolds. Due to special conformation, lncRNAs serve as platforms to collect relevant components including proteins and RNAs [3,9].

As an emerging field, more and more lncRNAs have been discovered by a wide use of sequencing techniques such as next-generation sequencing (NGS) and RNA sequencing (RNA-Seq) [10]. Numerous lncRNAs have been reported to be involved in different types of cancers including gastric cancer (GC) [11,12]. Meanwhile, LncRNA H19 has been considered as a vital player in cancer development, not only because the alteration of H19 expression is frequently observed, but also it actively participates in a wealth of malignancies and almost all stages of tumorigenesis [13].

H19 is a transcript from *H19*/*IGF2* genomic imprinted cluster which is located on chromosome 11p15.5. This imprinted gene locus also contains *IGF2* (insulin-like growth factor-II) gene. H19 is transcribed from the maternally-inherited chromosome, and IGF2 is from the paternally-inherited one, indicating the involvement of H19 in embryonic growth and development [14]. IGF2 has multiple functions in many biological processes, such as promoting cell growth [15]. Besides H19, there are still other transcripts from *H19*/*IGF2* locus including HOTS (H19 opposite tumor suppressor), 91H, PIHit and miR-675 [16]. *H19* gene, containing five exons, is the first imprinted lncRNA gene identified [17]. It is transcribed by polymerase II similar as mRNA except lacking a common open reading frame. Moreover, both sequence and secondary structure of H19 show a great extent of conservations among mammals, which may be consistent with its universal functions [18]. Due to alternative splicing, H19 has two main isoforms: one minor variant is without part of exon 1, which plays an important role during embryonic development for it is detected in human embryonic and placental tissues; the other one, lacking exon 4, is also shown with a potential effect on development [19,20].

Dysregulation of H19 has been reported in various kinds of tumorigenesis. However, in gastric carcinogenesis, the functional role of H19 has yet to be fully investigated. GC is one of the most malignant cancers around the world which causes thousands of deaths each year [21]. According to WHO statistics, it was the fourth most common cancer in the world in 2012 and occurs frequently in Asia, especially in China (approximately half of the world cases) [22]. GC is a complex disease and there are many outward factors that lie behind. Infection of *Helicobacter pylori* (*H. pylori*) is the main potential risk factor of GC, which contributes to more than 80% cases [23]. Other reasons including high-salt and low-vegetable diet, smoking, family history, and EBV infection also drive GC [24]. Although many advanced diagnostic and surgical approaches for treatment of GC have been set up, its overall survival rate is still low, partially owing to poorly understood molecular mechanisms [24]. Thus, in this review, we will comprehensively summarize the physiological role of H19 and its dysregulation in tumorigenesis, especially in GC.

## 2. Physiological Role of H19

As a parentally imprinted gene, expression of H19 shows a dynamic way during mouse and human embryo development. It starts from the blastocyst stage and reaches a high level in the tissues of endodermal, mesodermal and ectodermal origins [25]. However, once the baby is born, H19 expression will be inhibited in most of mammalian tissues [26,27]. A high accumulation of H19 has been detected in mature skeletal muscle of both mice and human, possibly due to enhanced RNA stabilization during muscle cell differentiation [28]. Moreover, a previous study also suggested that a high level of H19 was observed in primary human articular chondrocytes which was regulated by SOX9 [29].

DNA methylation status at imprinting control regions (ICRs) within *H19* promoter has been considered as the predominant controller of H19 expression during mammalian development [30]. Alterations of DNA methylation status in *H19* gene caused by genome-wide epigenetic reprogramming, including DNA methylation, methylation maintenance and DNA demethylation during mammalian life cycle, are mainly responsible for the dynamic expression pattern of *H19* gene [31].

Owing to a special and evolutionary-conserved secondary structure, the main functional pattern of H19 is to recruit protein or miRNA factors via related binding sites [32], while another pattern of H19 to exert its function is through H19/miR675 axis. The following sections describe the biological processes in which H19 is involved via either H19/miR-675 axis or associations with other partners.

### 2.1. H19/miR-675 Axis

Besides serving as an independent lncRNA, H19 is also the primary precursor of miR-675 and determines the level of this miRNA to a certain extent [33]. Since miR-675 has multiple targets in diverse signaling pathways, H19 is able to regulate a number of biological processes via miR-675. For example, it was reported that H19/miR-675 axis promoted skeletal muscle differentiation through decreasing Smad1, Smad5 and Cdc6 [34]. In addition, by targeting transforming growth factor-β1 (TGF-β1) and histone deacetylase 4/5 (HDAC4/5), H19/miR-675 axis facilitated osteoblast differentiation [35].

### 2.2. HuR (Human Antigen R)

Previous studies have demonstrated that H19/miR-675 axis was critical during mammalian development [14,36]. One of the mechanisms is through the association between H19 and HuR, a typical RNA binding protein [37]. H19–HuR interaction has been proven to inhibit the processing of miR-675 from H19 in placenta at Drosha stage, which decreased miR-675 and suppressed placental growth [37]. Silencing of H19 and miR-675 resulted in placental overgrowth in a mouse model and the dynamic expression of HuR closely regulated the fluctuant processing of miR-675 during gestation [37].

### 2.3. KSRP (RNA Binding Protein K Homology-Type Splicing Regulatory Protein)

KSRP, an RNA binding protein, binds to the AU-rich elements (ARE) of mRNA and makes mRNA decay [38]. KSRP also facilitates the maturation of a set of miRNAs by interacting with their precursors [39]. It was reported that KSRP interacted with H19 in the C2C12 cells. This interaction could be abrogated by the activation of AKT signaling pathway, and KSRP was released to bind with and degrade myogenin mRNA as well as other relative labile mRNAs during myogenic differentiation [40].

### 2.4. IMP-1 (IGF-II mRNA-Binding Protein 1) and PTBP1 (Polypyrimidine Tract-Binding Protein 1)

IMP-1 belongs to VICKZ family of zipcode-binding proteins. Members in this family interplay with mRNAs to affect cell proliferation and migration [41]. IMP-1 was proven to bind to H19 and IGF-II mRNA physically in a sequential and cooperative way through a series of in vitro experiments including electrophoretic mobility-shift assay (EMSA), equilibrium and kinetic assay. This well-ordered formation of protein–RNA complex favors final ribonuleoprotein (RNP) particle stability, which may be indispensable for storage, process and degradation of RNA transcripts [42]. It was further illustrated by an experiment conducted in mouse NIH3T3 embryo fibroblasts that the binding between IMP and H19 contributed to H19 subcellular localization in cytoplasm, which might play a crucial role during mammal development [43].

PTBP1, another RNA binding protein regulating RNA metabolism, is also one of the H19 interactive proteins identified by UV-crosslinking [43]. However, the function of H19-PTBP1 is still unknown.

### 2.5. HnRNP U (Heterogeneous Nuclear Ribonucleoprotein U)

As a ubiquitously existing ribonucleoprotein, hnRNP U has been characterized as an interactive protein of several lncRNAs such as Xist and PANDA [44,45]. It has been reported that, via associating with hnRNP U, H19 repressed RNA Pol II-mediated global transcription by inhibiting the phosphorylation of RNA Pol II carboxy-terminal domain (CTD) at Ser5 in HCC cells [46,47]. Since RNA Pol II-mediated global transcription also plays a role under pathological conditions, this H19/HnRNP U system is highly expected to serve as a novel therapeutic target in certain human diseases.

### 2.6. MBD1 (Methyl-CpG–Binding Domain Protein 1)

*H19* gene, which belongs to imprinted gene network (IGN), controls the expression of other eight imprinted genes within this network, such as *IGF2*, *SLC38A4*, *DCN*, *DLK1*, *PEG1*, *GTL2*, *CDKN1C* and *IGF2R*. All of them were reported to regulate embryonic growth in mouse and human [48]. When *H19* gene is deleted, these targeted genes are up-regulated, and subsequently an overgrowth phenotype of mice appears [49]. In general, expression of imprinted genes are predominantly determined by epigenetic modifications [50]. There was a report indicating that H19 repressed these imprinting genes via interacting with MBD1 (methyl-CpG-binding domain protein 1) in primary MEFs. MBD1 is one of the members of a nuclear protein family; all of these members, including MECP2 and MBD1-4, contain the same methyl-CpG binding domain (MBD) [51]. MBD1 binds specifically to methylated DNA domains and recruits methylation related enzymes to modulate gene expression [51]. MBD1 was suggested to be driven by H19 to differentially methylated regions (DMRs) of targeted imprinted genes (*IGF2*, *SLC38A4* and *PEG1*) to induce H3K9me3 modifications, so as to regulate those genes expression [51].

### 2.7. PRC2 (Polycomb Repressive Complex 2)

PRC2 has been predicted to be associated with an estimated 20% lncRNAs [52], many of which have been validated, such as HOTAIR [53], MALAT1 [3] and MEG3 [54,55]. H19 has also been identified as a member of PRC2-associating transcriptome. As a key chromosome epigenetic regulation complex which consists of core components (EZH2/EZH1, EED, SUZ12, and RBBP4/6) and supplementary ones (PCLS and JARID2), PRC2 keeps transcriptional repression by methylating H3K27me2/3 [56].

### 2.8. SAHH (S-Adenosylhomocysteine Hydrolase)

SAHH is the only enzyme that catalyzes the hydrolysis of SAH, an inhibitor of SAM-dependent methyltransferases (*S*-adenosylmethionine). Importantly, SAHH participates in SAM-dependent gene methylation in many biological processes [57]. It was identified as one of the interactive proteins of H19 in ribonucleoprotein complexes in HEK293 cells. SAHH bounded to the U-rich element in 3’-terminus of H19, which suppressed SAHH enzyme activity. This was examined in both skeletal muscle cells and myoblasts derived myotubes in mice model [58]. Silencing of H19 led to an increased activity of SAHH and further promoted DNMT3B-dependent gene methylation [58]. This effect of H19-SAHH complex may extend to global biological processes according to genome-wide methylation profiling after H19 silencing [58].

### 2.9. Let-7 Family

Kallen et al. have already proven that H19 absorbed let-7 family members through conserved binding sites and interacted with miRNA ribonucleoprotein complexes, leading to inhibition of let-7 activity and regulation of let-7 downstream effectors, such as Dicer, HMAG2 and IGF2, therefore, mouse muscle differentiation was slowed down and a precocious differentiation was prevented [59]. In another case, H19 serves as an upstream suppressor of let-7 bioavailability. Loss of H19 in muscle of both human subjects with type-2 diabetes and mice with insulin resistant resulted in an increase of let-7 bioavailability and reduced expression of let-7 targets, including insulin receptor (INSR) and Lipoprotein lipase (LPL), which were mainly responsible for the following impaired glucose uptake [60].

### 2.10. miR-106a

A novel approach named miRNA crosslinking and immunoprecipitation (miR-CLIP) developed by Jochen Imig and his colleagues was applied to identify lncRNA-miRNA interactions. The results illustrated that H19-miR-106a interaction occurred in both Hela cells and myoblasts, where H19 functioned to regulate the level of miR-106a, which was crucial during myoblast differentiation [61].

### 2.11. Summary of H19 Binding Partners

**Figure 1:**
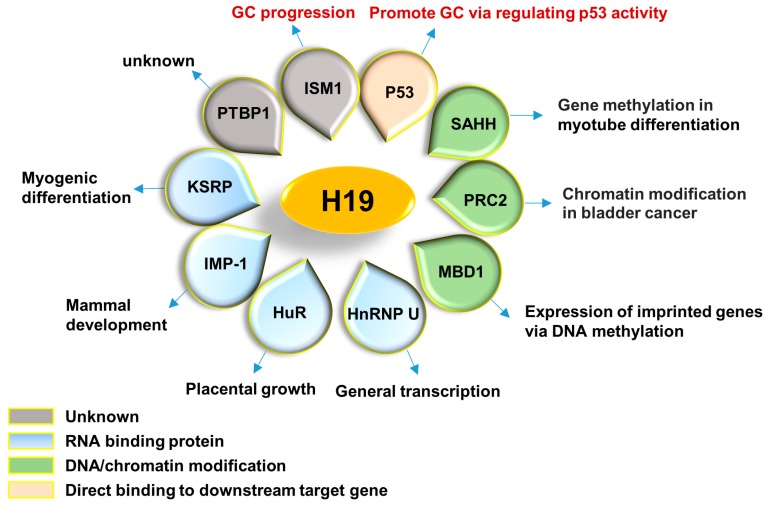
H19 associated proteins. Different associated proteins of H19 reported up to date are shown. These proteins are involved in various biological processes as indicated in the outward cycle. Most of them are RNA binding proteins (KSRP, IMP-1, HuR and HnRNP U) and DNA/chromatin modification factors (SAHH, PRC2 and MBD1), suggesting of a crucial role of H19 on gene expression. P53 and ISM1 have been identified as associated proteins of H19 in GC. PTBP1, another RNA binding protein, has also identified as one of H19 interaction proteins with unclear function. GC, gastric cancer.

**Figure 2:**
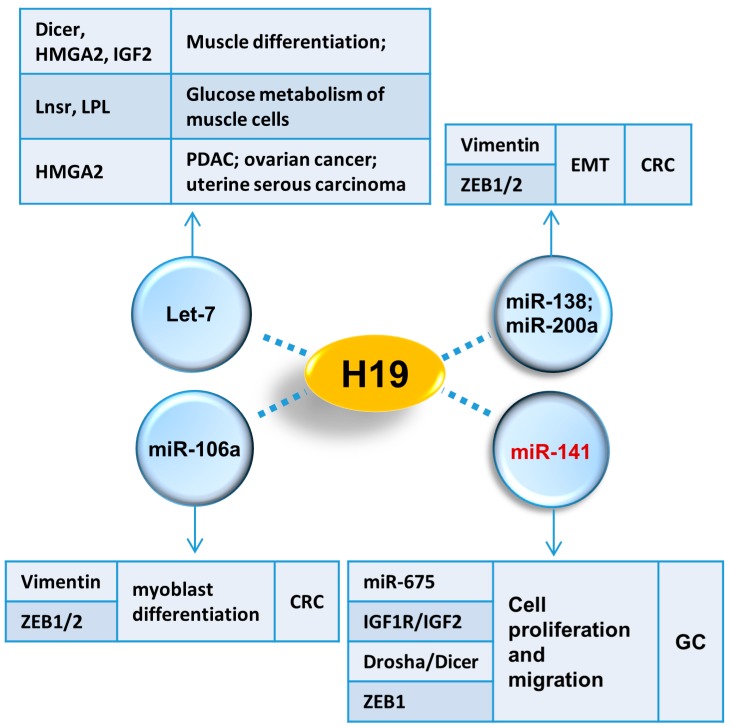
H19 interactive miRNAs. Different associated miRNAs of H19 are demonstrated. These four miRNAs are involved in several kinds of cancers and development via targeting functional factors as indicated in tables. In GC, H19 associates with miR-141 and further affects downstream targets: miR-675, IGF1R, IGF2, Drosha, Dicer and ZEB1. EMT, epithelial–mesenchymal transition; CRC, colorectal cancer; GC, gastric cancer.

H19 is capable of being folded into a special secondary structure, which allows it to serve as a platform and collect relative proteins [62]. In addition, these proteins are actively involved in a wide variety of physiological and pathological processes such as RNA metabolism, gene transcription, epigenetic modification, skeletal muscle differentiation and tumor development, suggesting a diversified function of H19. All of the interactive proteins with H19 are summarized in Figure 1. Meanwhile, miRNAs are another group of partners that are essential for lncRNAs to exert their functions. In fact, miRNAs themselves are crucial modulators of gene expression transcriptionally or post-transcriptionally via targeting to mRNAs [63]. LncRNAs usually function as a “sponge” to disable miRNAs [64], while sometimes, lncRNAs and miRNAs can also regulate expression of each other [65,66]. miRNAs which interact with H19 are summarized in Figure 2.

## 3. The Role of H19 in Tumorigenesis

H19 is one of the well-studied lncRNAs during tumorigenesis [67]. It was first identified as a tumor suppressor [68]. However, accumulating evidence showed that H19 was significantly increased in several tumors as an oncogene [13,69]. Whether functional role of H19 is tumor suppressive or oncogenic is still controversy, and it might depend on different cancer types, development stages and molecular background to a large extent [70,71]. Abnormal expression of H19 has been discovered in a bulk of cancers [72,73,74], including breast cancer [75], lung cancer [71], cervical cancer [76] and bladder cancer [69]. The underneath molecular mechanisms are largely dependent on the partners that H19 works with.

### 3.1. H19/miR-675 Axis

H19/miR-675 axis was illustrated to play essential roles in cancers through targeting oncogenic or tumor suppressive factors [13,77]. H19-derived miR-675 directly targeted and decreased c-Cbl and Cbl-b in breast cancer cells [78]. Loss of c-Cbl and Cbl-b further induced the expression of tyrosine kinase receptors and the activation of AKT and ERK signaling pathway, and therefore, enhanced proliferation and metastasis of breast cancer cell both in vitro and in vivo [78]. Retinoblastoma protein (Rb), a tumor suppressor, was verified as a target of miR-675 both in colorectal cancer and AFP-secreting hepatocellular carcinoma [79,80]. Other targets of miR-675 in different tumors contain: Twist 1 (a key mediator in EMT) in hepatocellular cancer [80], Cadherin 13 (a member of cadherin subfamily) in glioma [81], G protein-coupled receptor (GPR55) in non-small cell lung cancer [82] and TGFBI (an extracellular matrix protein) in prostate cancer [83]. miR-675 was also demonstrated to play an important role in bladder cancer cell growth via modulating p53 level, although p53 was not a direct target of miR-675 [84].

### 3.2. H19/let-7 Axis

H19/let-7 axis functioned as an oncogene in some tumors. For example, H19 promoted invasion and migration of pancreatic ductal adenocarcinoma (PDAC) cells via stopping let-7 from targeting HMGA2 [85]. With the similar molecular mechanism, H19 increased metastatic ability of pancreatic cancer as well as ovarian cancer and uterine serous carcinoma cells.

### 3.3. p53

P53 is a well-known tumor suppressor and lies in the center of multiple cancer-related signaling pathways [86]. Meanwhile, H19-derived miR-675 has been shown to promote bladder cancer cell proliferation via repressing p53 expression [84]. On the other hand, the fact that the binding sites for p53 located within H19 promoter was identified, which allowed p53 negatively regulate expression of H19 in tumor cells [87]. In addition, H19 expression was increased sharply in many cancer cell lines of liver, bladder, lung, ovarian and breast under the condition of hypoxic stress, which occurs commonly in tumor development [62]. During hypoxia, p53 increases H19 through activating HIF signaling pathway [62].

### 3.4. IGF2

IGF2 promotes proliferation in several cancer types and serves as an anti-apoptotic agent [88]. H19 and IGF2 expression is activated by a common enhancer, it is also a common binding partner both H19 and IGF2 compete for [89]. Thus, excessive H19 usually induces a sharp decrease of IGF2, whereas deletion of H19 results in IGF2 overexpression [89]. Interestingly, another lncRNA from the same gene locus named 91H, which is the antisense of H19, also regulates IGF2 expression in esophageal squamous cell carcinoma and breast cancer [90,91].

### 3.5. PRC2

In bladder cancer, H19 has been proven to interact with PRC2 through EZH2, one of the core components of PRC2 complex by RNA immunoprecipitation assay [69]. Binding to H19 further facilitates EZH2 recruitment to the promoters of E-cadherin (cell metastasis repressor) and Nkd1 (inhibitor of Wnt/β-catenin signaling). Therefore, expressions of E-cadherin and Nkd1 are suppressed leading to enhanced metastasis and invasion of bladder cancer cells [69]. In addition, it has been proven that H19/EZH2 complex is a vital regulator during mammary tumorigenesis [75].

### 3.6. miR-200a and miR-138

In colorectal cancer cells, miR-200a and miR-138 have been verified as interactive miRNAs of H19 by Liang et al. [92]. This interaction impairs bioavailability of miR-200a and miR-138 to their mRNA targets (Vimentin, ZEB1 and ZEB2). Vimentin and ZEB1/2 are crucial mediators of epithelial to mesenchymal transition (EMT) in colon cancer. Excessive H19 functioned as a “molecular sponge” to absorb miR-200a and miR-138, and promoted EMT of colorectal cancer cell lines [92].

## 4. Dysregulation of H19 in Gastric Carcinogenesis

GC is one of the most malignant and common tumors around the world, and there have already been plenty of studies illustrating that H19 is significantly up-regulated in both GC tissues and cell lines, and is positively correlated with poor prognosis.

In a previous study, H19 was shown as one of the most increased lncRNAs with a ~8.91-fold change in human primary GC tumors comparing with non-tumor tissues [12]. Additionally, Li et al. also identified some potential lncRNAs that expressed differently between gastric tumors and normal tissues through screening a 74 GC patients cohort [93], among which, H19 was selected because of a remarkable increased level in GC tissues. Moreover, several GC cell lines, such as MKN45, BGC-823 and AGS exhibited a high expression level of H19 [93,94]. Ectopic expression of H19 could promote proliferation, migration and invasion of GC cells, whereas silencing of H19 resulted in cell apoptosis [88,89,90,91]. Intriguingly, H19 was reported to be not only up-regulated in GC tissues but also in the plasma of GC patients. Circulating H19 in plasma could serve as a potential biomarker for GC clinical diagnosis at early stage [95]. Similarly, in GC, H19 behaved diversely with different companions.

### 4.1. H19/miR-675 Axis

**Figure 3:**
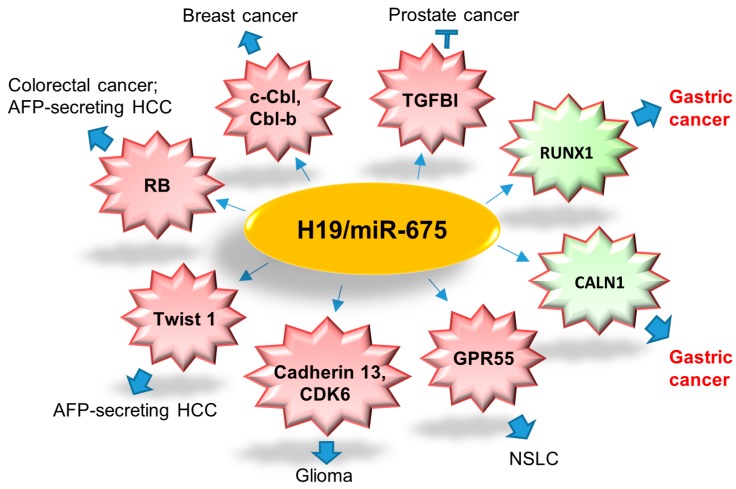
Summary of direct targets of H19/miR-675 axis in different cancer types. H19/miR-675 suppresses prostate cancer metastasis via decreasing TGFB1; enhances tumorigenesis and metastasis of breast cancer by downregulating c-Cbl and Cbl-b; promotes colorectal cancer via decreasing tumor suppressor, RB; mediates AFP-secreting HCC as oncogene via decreasing RB and Twist 1; increases tumorigenesis potential of glioma via targeting Cadherin 13 and CDK6; modulates NSCLC development as a tumor suppressor via targeting GPR55; promotes gastric tumorigenesis through targeting RUNX1 and CALN1. RB, retinoblastoma protein; HCC, hepatocellular carcinoma; NSCLC, non-small cell lung carcinoma.

Both H19 and miR-675 are up-regulated in primary GC when compared with noncancerous tissues [93]. H19/miR-675 axis promoted proliferation, migration, invasion of GC cells and subcutaneous tumors in nude mice [93,96]. Runt Domain Transcription Factor1 (RUNX1), a member of Runt-related (RUNX) gene family and also a crucial tumor suppressor, has been identified as a direct target of miR-675 in GC [96]. H19/miR-675 axis was shown to enhance GC cell growth via inhibiting RUNX1 and activating Akt/mTOR signaling pathway [96,97]. Calneuron 1(CALN1) is another target of miR-675 in GC [93]. All the targets of miRNA-675 are summarized in Figure 3.

### 4.2. p53

Researchers have found that physical binding between H19 and p53 served as both effector and regulator of p53 in GC [94]. Overexpression of H19 partially inactivated p53 activity and suppressed expression of Bax, which highly induced cell proliferation in GC [94].

### 4.3. IGF2

As parts of imprinted gene cluster, expression of H19 and IGF2 are well controlled by genomic imprinting [98]. Loss of imprinting (LOI) of H19–IGF2 locus frequently occurred in a variety of cancers [99,100]. Yu et al. examined LOI of IGF2 and H19 in 89 GC patients and found that the positive rate of IGF2 and H19 LOI in GC tissues were 45% and 8.6%, respectively. Moreover, GC in corpus has a high feasibility of IGF2 LOI compared with that in antrum [101].

### 4.4. Isthmin1 (ISM1)

ISM1, usually known as an angiogenesis inhibitor, has been identified as another H19 binding protein in MKN45 cell line. Meanwhile, ISM1 expression is regulated by H19 [93]. Although detailed biological function of the association between H19 and ISM1 has not been illustrated, it has been proposed that this association regulates GC cell growth and mobility because ISM1 regulates surviving and apoptosis in many tumor cells [102].

### 4.5. miR-141

H19 expression is also regulated by a series of molecules during GC development. Similar to proteins, expression of H19 is controlled by certain transcription factors such as c-Myc [101]. In GC cell lines, SGC-7901 and MKN45, miR-141 has been shown to suppress H19 expression by binding to it and further led to H19 degradation. After ectopic expression of miR-141, targets of H19 including miR-675, IGF1 and IGF2 are all declined. Meanwhile, H19 modulates Drosha and Dicer as well as ZEB1, a target of miR-141 [103].

### 4.6. Other Companions

Besides these already-validated interactive proteins and miRNA described above, there are still some partners predicated by several computational methods based on some well-established RNA-binding proteins (RBP) datasets such as StarBase version 2.0 (http://starbase.sysu.edu.cn/) and LNCipedia (http://www.lncipedia.org/) [104]. StarBase v2.0 shows that H19 is predicted to interact with eIF4AIII, DGCR8 and FU, as well as numerous miRNAs.

## 5. Conclusions and Future Directions

To our knowledge, despite its emerging and complicated biological functions, H19 exerts its functions primarily only through two direct mechanisms: producing miR-675 as its primary precursor or interacting with diversified partners including proteins and miRNAs. Although the currently identified targets of miR-675 seem to display a scattered distribution, most of them participate in growth, differentiation and different stages of tumor development, which also suggests an important position of H19 in growth-related gene networks. Targeting either H19 or the production of miR-675 may lead to the discovery of putative therapeutic methods for some aberrant-miR-675-induced human diseases.

**Figure 4:**
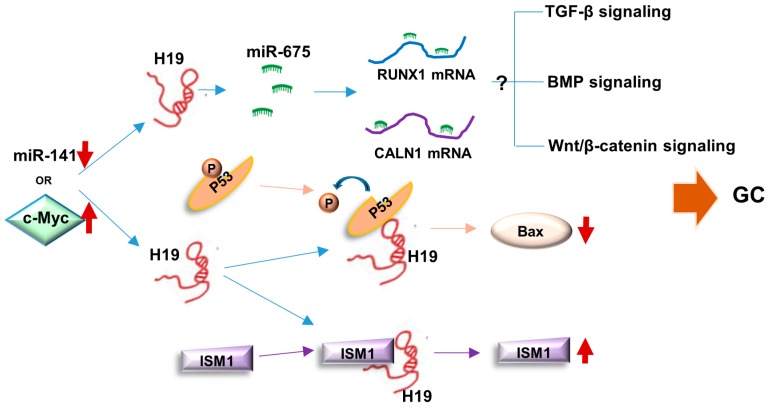
Summary of molecular mechanisms of H19 in GC development. On the one hand, miR-675 derived from H19 can target and degrade RUNX1 and CALN1 mRNA to enhance GC development via influencing potential signaling pathways. On the other hand, H19 binds with p53 to inhibit its activity and decrease BAX. H19 can also bind to ISM1 to induce ISM1 stabilization. The downregulation of miR-141 and c-Myc activation can up-regulate H19 expression in gastric tumorigenesis. GC, gastric cancer; up arrow, upregulation; down arrow, downregulation.

Direct interacting with special partners mechanically under certain physiological and pathological conditions is the most common characteristic of lncRNA [105]. We have summarized binding proteins and miRNAs of H19 in a variety of cell context, and those related to GC were highlighted. The close interactive network of H19 in GC development is shown in Figure 4. In spite of the fact that only p53 and ISM1 have been identified as H19 interaction proteins in GC, other associated proteins in diverse biological processes of different cancers might provide valuable clues for a further investigation. The network of H19 interactive proteins might be explored and an overall view of H19 functions in GC would be elucidated. Moreover, it is also important for researchers to find out the reasons why H19 switches its associative protein partners in different development periods, physiological statuses or clinical diseases.

Although many efforts have been made to investigate the associating factors of H19 and only a handful of proteins and miRNAs have been identified, still very limited is known in regards to the molecular mechanisms and biological effects of the interactions between H19 and its binding partners. More efforts should be made towards this direction, especially in tumor research. Currently, a phase I/IIa clinical trial of H19-based constructs (BC-H19/DTA-H19) in bladder cancer has already indicated a response rate of 66% [106]. The results may provide novel study angles for clinical treatment of cancer patients.
